# Porous-starch-encapsulated quercetin remodels the gut microbiota and elevates short-chain fatty acids, alleviating hyperglycaemia-associated phenotypes in T2DM mice

**DOI:** 10.3389/fnut.2026.1851659

**Published:** 2026-09-09

**Authors:** Peipei Kang, Yongyan Zheng, Shuman Song, Yiyin Gao, Yuanhong Song, Huayang Wang, Tao Jiang, Xinran Xiang

**Affiliations:** 1 Luoyang Key Laboratory of Zebrafish Toxicology Research, Luoyang Key Laboratory of Transplantation and Immunological Studies for Haematological Diseases, Department of Clinical Laboratory, The First Affiliated Hospital, and College of Clinical Medicine of Henan University of Science and Technology, Luoyang, China; 2 Jiangsu Key Laboratory of Huaiyang Food Safety and Nutrition Function Evaluation, Jiangsu Collaborative Innovation Center of Regional Modern Agriculture & Environmental Protection, School of Life Science, Huaiyin Normal University, Huaian, China

**Keywords:** gut microbiota, hyperglycemia, porous-starch-encapsulated, quercetin, type 2 diabetes mellitus

## Abstract

**Introduction:**

Quercetin (QUE), a plant-derived flavonoid abundant in many vegetables, has been implicated in glycaemic regulation; however, its application in functional foods is limited by poor stability during upper-gastrointestinal transit and low oral bioavailability. This study aimed to develop a porous-starch-based delivery system to improve the bioavailability and metabolic benefits of QUE.

**Methods:**

Porous-starch-encapsulated quercetin microspheres (PSQ) were engineered using corn-derived porous starch as a carrier. The *in vitro* release behavior of PSQ was evaluated under simulated gastric and intestinal conditions. The metabolic effects of PSQ were further investigated in a type 2 diabetes mellitus (T2DM) mouse model by comparing PSQ with free QUE. Glucose metabolism, lipid metabolism, gut microbiota composition, and caecal short-chain fatty acids (SCFAs) were systematically analyzed.

**Results:**

In vitro, PSQ showed restrained release under gastric-like conditions but enhanced release under intestinal-like conditions, indicating reduced premature release and improved intestinal-phase availability. In T2DM mice, PSQ administration improved body weight, fasting blood glucose (FBG), oral glucose tolerance test (OGTT), serum glycated protein (GSP), and HOMA-derived indices of islet function, while partially alleviating serum and hepatic lipid abnormalities. The higher PSQ dose exhibited stronger effects than the lower dose and free QUE. Microbiota analysis revealed PSQ-associated enrichment of beneficial metabolic-related genera, including *Lactobacillus, Adlercreutzia*, and *Limosilactobacillus*, together with depletion of taxa such as *Lawsonibacter* and *Bilophila*. These microbial alterations were accompanied by increased caecal SCFA levels, which were significantly correlated with glycaemic and biochemical parameters. Correlation-network analysis identified *Turicimonas* and *Muribaculum* as potential microbial signatures associated with metabolic regulation.

**Discussion:**

Porous-starch encapsulation provides partial gastric protection and enhances intestinal availability of quercetin, thereby potentiating its metabolic benefits in T2DM mice. The improved metabolic outcomes were accompanied by coordinated modulation of gut microbiota and SCFA production, supporting PSQ as a promising strategy for developing QUE-based functional food interventions.

## Highlights

- PSQ advances a food-compatible encapsulation strategy to overcome the defect of low bioavailability in a widely studied flavonoid.
- PSQ demonstrates clear hypoglycemic efficacy *in vivo* with an explicit dose comparison.
- PSQ links functional outcomes to coordinated changes in microbiota and SCFAs.

## 1 Introduction

Diabetes mellitus is an endocrine metabolic disorder defined by persistent hyperglycaemia, most commonly arising from insufficient insulin secretion and/or impaired insulin action (1–3). In recent years, alongside rapid socioeconomic changes and shifts in lifestyle, the global burden of diabetes has continued to rise (4). According to the International Diabetes Federation (IDF), an estimated 463 million adults aged 20–79 years were living with diabetes in 2019; China alone accounted for approximately 116.4 million cases, about one quarter of the global total, ranking first worldwide (5). By 2021, the number of affected adults had increased to ~537 million globally and to 140.9 million in China, which remained the country with the largest patient population (https://www.diabetesatlas.org) (5). Over this 2-year interval, the global caseload expanded by ~15.98%, whereas China experienced a larger increase (~21.05%); notably, type 2 diabetes mellitus (T2DM) represents more than 90% of all diabetes cases. As T2DM progresses, sustained hyperglycaemia drives chronic, multi-organ injury, including the liver, intestine and cardio-cerebrovascular system, and promotes complications that can culminate in largely irreversible health consequences (6). Accordingly, developing effective strategies to control hyperglycaemia and delineating its underlying biology in T2DM remain critical for improving patient outcomes and mitigating the overall health burden (7, 8).

The gut microbiota constitutes an integral component of the host metabolic network and exerts broad control over physiological homeostasis. Accumulating evidence indicates that reductions in microbial diversity and shifts in community abundance are tightly linked to the onset and progression of T2DM (9). Consistent with this view, The previous studies suggested that disturbances in glucose metabolism coincide with a remodeling of gut microbial architecture and track with T2DM-related phenotypes (10, 11); at the genus level, changes in *Lactobacillus, Lachnoclostridium* and *Desulfovibrio* were significantly associated with multiple serum and hepatic biochemical indices in T2DM mice, including total cholesterol (TC), triglycerides (TG), low-density lipoprotein cholesterol (LDL-c) and high-density lipoprotein cholesterol (HDL-c) (11). Functional interventions further support a causal contribution: Ai et al. (12) reported that a *Lactobacillus*-fermented rice bran preparation enhanced glucose consumption in insulin-resistant HepG2 cells, reduced intracellular lipid accumulation, and decreased glycaemia and lipidaemia while improving antioxidant capacity in T2DM mice. Human case control profiling similarly shows that *Lactobacillus* and *Bifidobacterium* are depleted in individuals with T2DM relative to healthy controls, implicating these taxa as negatively associated with disease progression (13). Beyond compositional shifts, dysbiosis can compromise intestinal barrier integrity, potentially accelerating T2DM progression and propagating multi-organ injury through downstream metabolic and inflammatory pathways (14). For example, Song et al. (15) suggested a potential causal relationship between increased *Lachnoclostridium* abundance and T2DM risk, whereas Li et al. (16) showed that an ethanol extract of *Inonotus hispidus* reduced *Desulfovibrio* abundance and improved barrier-related phenotypes in T2DM mice. Collectively, a dysbiotic signature, characterized by depletion of beneficial taxa and expansion of potentially deleterious microbes, has emerged as a plausible and actionable axis for mechanistic interrogation and intervention in T2DM.

Quercetin (QUE) is a ubiquitous flavonoid (3,3′,4′,5,7-pentahydroxyflavone) that typically appears as a yellow crystalline powder and is abundant in dietary sources such as apples, onions, tea, red wine and berries (17). Prior studies suggest that QUE may ameliorate dysglycaemia through multi-layered mechanisms, including lowering blood glucose, alleviating insulin resistance, restoring intestinal barrier integrity, remodeling the gut microbiota, and reshaping microbial metabolite profiles in db/db mice (18). Despite these promising bioactivities, industrialization is hindered by a clear pharmaceutic bottleneck: QUE displays limited stability during oral transit, is sensitive to light, heat and metal ions, and readily undergoes oxidative degradation, resulting in low oral bioavailability and restricting its therapeutic potential in T2DM. The central challenge therefore is not simply identifying bioactivity, but engineering an oral delivery strategy that preserves QUE through the upper gastrointestinal tract, enables controlled intestinal release, and allows its microbiota- and metabolite-linked effects to manifest robustly *in vivo*.

**Figure 1:**
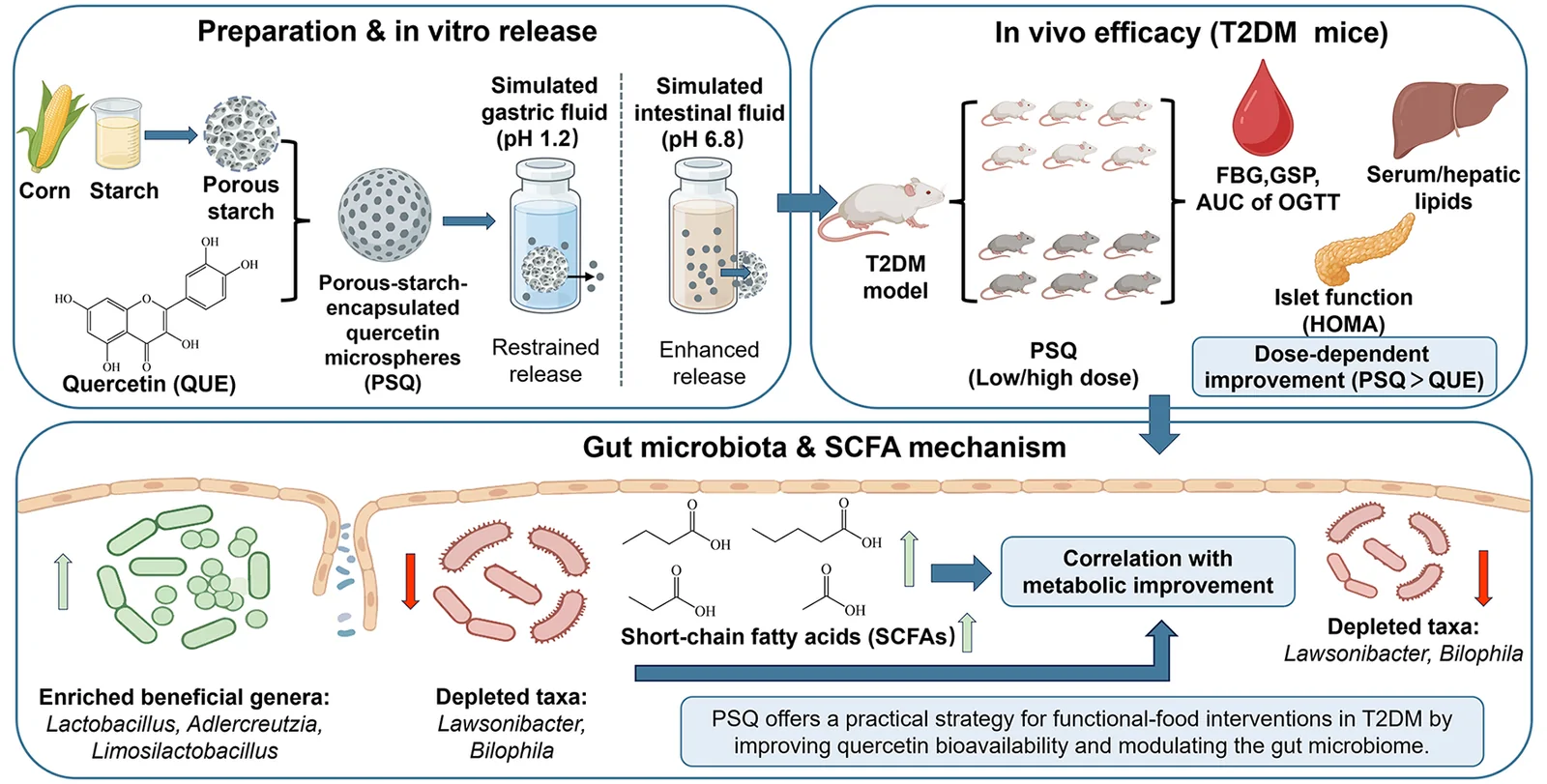
Preparation, release, hypoglycemic effects, and gut microbiota regulation of PSQ microspheres.

Efforts to enhance the oral performance of QUE have fuelled the development of multiple delivery platforms, including solid dispersions, liposomes and microsphere-based carriers. Among these, microspheres have attracted sustained interest because they can be manufactured with comparatively tractable processes, exhibit favorable physicochemical stability, and improve the *in vivo* utilization of lipophilic bioactives (19, 20). A recurrent material choice for such systems is porous starch (PS), which is typically produced by mild enzymatic etching of native corn starch to generate a highly porous architecture with large specific surface area and strong adsorption capacity, while retaining a favorable safety profile (21). Relative to more elaborate carrier materials, PS offers a pragmatic combination of attributes, broad availability, low cost, biodegradability and low toxicity risk, together with a pore-rich structure that enables efficient loading without substantially perturbing the intrinsic physicochemical properties of the encapsulated compound. Importantly, the progressive digestion of starch *in vivo* provides a mechanistic basis for sustained release and intestinal-stage delivery, which may in turn improve bioavailability and strengthen the reproducibility of downstream biological effects. Accordingly, PS microspheres have been positioned as a versatile delivery modality across food and pharmaceutical contexts (22, 23). Building on this rationale, we formulated porous-starch-encapsulated quercetin microspheres (PSQ), which provide partial gastric protection and enhanced intestinal bioavailability of QUE, and systematically evaluated, in a T2DM mouse model, whether this delivery strategy ameliorates hyperglycaemic phenotypes and remodels gut microbial community, providing experimental evidence for improving QUE utilization and advancing mechanistic understanding of its antihyperglycaemic potential (Figure 1).

## 2 Materials and methods

### 2.1 Preparation of QUE-loaded PS microspheres and *in vitro* release assay

QUE (100 mg) was dissolved in acetone (100 mL) under sonication until a clear solution was obtained. 200 mg of porous starch (PS; pore size: 0.3–2 μm, porosity: 50%, catalog no. abs2026, Shaanxi Aibeisi Biotechnology Co., Ltd., China) was then added, followed by further sonication at room temperature to ensure uniform dispersion. Regarding the source and characteristics of the porous starch: a certificate of analysis has been acquired from the supplier (Shaanxi Aibeisi Biotechnology Co., Ltd., China; Supplementary Table S1). The corn variety (Shaanke 9), the amylose-to-amylopectin ratio (23:77), and the enzymatic hydrolysis conditions, as follows: enzyme dosage of 1% (on a dry starch basis), α-amylase to glucoamylase mass ratio of 1:2, temperature of 55 °C, pH 5.0 phosphate–citrate buffer (0.2 mol/L Na_2_HPO_4_ and 0.1 mol/L citric acid, mixed to pH 5.0), and hydrolysis duration of 16 h with a stirring rate of 120 rpm. The enzyme activities were specified as α-amylase ≥ 100,000 U/g and glucoamylase ≥100,000 U/g (measured under the supplier's standard assay conditions at 40 °C, pH 4.6). The QUE concentration was quantified by high-performance liquid chromatography (HPLC, Agilent 1260 Infinity II, USA). Chromatographic separation was performed using a Kromasil C18 column (4.6 mm × 250 mm, 5 μm). The mobile phase consisted of methanol and 0.2% aqueous phosphoric acid at a volume ratio of 48:52. The flow rate was 1.0 mL/min, the detection wavelength was 360 nm, the column temperature was maintained at 30 °C, and the injection volume was 10 μL. PSQ and free QUE were analyzed in parallel under identical experimental conditions to compare their relative release profiles. Encapsulation efficiency (EE%) and drug loading capacity (DLC) for PSQ were calculated as follows: EE% = (total QUE–free QUE in supernatant)/total QUE added × 100%; DLC% = (total QUE–free QUE in supernatant)/weight of PSQ × 100%, where total QUE represents the initial weight of QUE, free QUE in supernatant represents the unencapsulated QUE weight in the supernatant, and weight of PSQ represents the total weight of the PSQ microspheres.

Simulated gastric fluid (SGF, pH 1.2) and simulated intestinal fluid (SIF, pH 6.8) were used as the release media for the *in vitro* release experiments. SGF was prepared by dissolving 2.0 g of NaCl and 3.2 g of pepsin, with an activity of 800–2,500 units/mg protein, in approximately 7.0 mL of concentrated hydrochloric acid and an appropriate volume of deionized water. The solution was diluted to a final volume of 1,000 mL, and its pH was adjusted to 1.2 using hydrochloric acid. SIF was prepared by dissolving 6.8 g of potassium dihydrogen phosphate in 250 mL of deionized water. The pH was adjusted to 6.8 using 0.1 mol/L sodium hydroxide, after which 10.0 g of trypsin was added, and the solution was diluted to a final volume of 1,000 mL (24). To maintain sink conditions, 0.5% Tween 80 was added to both release media. The *in vitro* release experiments for PSQ and free QUE were conducted at 37 °C under magnetic stirring at 100 rpm. At predetermined time points of 1, 2, 3, 4, 5, 6, 8, 12, and 24 h, 1.0 mL aliquots were withdrawn and immediately replaced with an equal volume of fresh release medium maintained at the same temperature. To quantitatively compare the overall release behavior of PSQ and free QUE in SGF and SIF, the area under the cumulative release-time curve (AUC) was calculated using the trapezoidal rule as follows Equation 1:


$$\begin{array}{l} AUC_{0-6h}=\sum_{i=1}^{n}\frac{\left(C_{i}+C_{i-1}\right)\times \left(t_{i}-t_{i-1}\right)}{2} \end{array}$$ (1)

where Ci represents the cumulative release rate (%) at time point i, and ti is the corresponding time (h).

### 2.2 Scanning electron microscopy and X-ray diffraction analysis

The apparent morphology of PSQ was observed by scanning electron microscopy (SEM). X-ray diffraction (XRD) analysis was performed according to the method described by Sinhmar et al. (25). The sample was uniformly packed into the recessed cavity of a dedicated sample holder, and the excess material was gently removed with a glass slide to obtain a flat and level surface. The prepared sample holder was subsequently placed in an X-ray diffractometer (SmartLab SE, Rigaku, Japan), and the corresponding XRD patterns were recorded. The instrument was operated using Cu Kα radiation with a wavelength of 0.154060 nm at a tube voltage of 40 kV and a tube current of 40 mA. Diffraction data were collected over a 2θ range of 5°-60° at a scanning rate of 2°/min.

### 2.3 Specific surface area and pore volume analysis

The specific surface area and pore volume of the samples were determined using an automated surface area and porosity analyzer based on low-temperature nitrogen adsorption-desorption. Approximately 0.06 g of each sample was degassed at 300 °C for 10 h prior to analysis. Nitrogen adsorption-desorption isotherms were recorded over a relative pressure (P/P_0_) range of 0.01–1.00. The specific surface area was calculated using the Brunauer–Emmett-Teller (BET) method, whereas the pore volume was determined using the Barrett–Joyner–Halenda (BJH) method.

### 2.4 Grain diameter distribution analysis

The grain diameter distributions of PS and PSQ were determined according to the method reported by Ge et al., (26) with minor modifications. Briefly, 0.5 g of PS or PSQ was dispersed in 10 mL of deionized water and ultrasonicated to obtain a homogeneous suspension. Deionized water was used as the dispersing medium, and the suspension was gradually introduced into the dispersion cell containing deionized water at a circulation speed of 2,000 rpm. Measurements were conducted over a grain diameter range of 0.02–2,000 μm. The refractive indices of water and the PS and PSQ particles were set to 1.33 and 1.53, respectively.

### 2.5 Animal experiment

42 healthy male Institute of Cancer Research (ICR) mice (Specific Pathogen-Free (SPF) grade; 4 weeks of age; 20 ± 3 g) were obtained from the Comparative Medicine Center of Yangzhou University. Animals were housed in a humidity barrier system under controlled conditions (24 ± 2 °C; 50 ± 10% relative humidity) with a 12 h light-dark cycle and *ad libitum* access to food and water. After a 1-week acclimation period, six mice were randomly assigned to the Normal group and maintained on a standard chow diet, whereas the remaining mice were fed a high-sugar/high-fat diet. The High-Sugar High-Fat (HSHF) diet was purchased from Wuxi Fanbo Biotechnology Co., Ltd. (catalog no. FB1040), which contained 58.8% standard feed, 15% sucrose, 15% lard, 1% cholesterol, 0.2% cholate, and 10% egg yolk. After 4 weeks, all high-fat-fed mice were subjected to streptozotocin (STZ) administration to induce T2DM: STZ was freshly prepared in 0.1 M citrate buffer (pH 4.5) and injected intraperitoneally at 35 mg/kg, three times per week. Normal mice received citrate buffer alone at an equivalent dose/volume. Forty-eight hours after the third STZ injection, mice were fasted for 12 h (with free access to water), and fasting blood glucose (FBG) was measured from tail-vein blood; mice with FBG ≥11.1 mmol/L were considered diabetic. In total, 36 mice were subjected to STZ injection, of which 30 met the inclusion criteria and were randomized into the following groups: Model (T2DM), PSQ-L (50 mg/kg·d), PSQ-H (100 mg/kg·d), QUE (100 mg/kg·d) and MET (Metformin hydrochloride; 100 mg/kg·d), the remaining six mice that failed to meet the FBG criteria were excluded from the study. For randomization, a computer-generated random number table (Microsoft Excel, RAND function) was used. To minimize potential baseline differences in glycaemia among groups, stratified randomization based on FBG levels was performed: mice were first divided into three FBG strata (11.1–13.0, 13.1–15.0, and >15.0 mmol/L), followed by random allocation to the five treatment groups within each stratum. Treatments were administered by oral gavage for 4 weeks. General health status was monitored daily, and water/food intake was recorded; body weight and FBG were measured weekly. At week 4, an oral glucose tolerance test (OGTT) was performed after a 12 h fast. 0 h blood glucose level (G: 0 h) was measured, followed by oral glucose administration (2 g/kg body weight), and blood glucose was monitored at 0.5, 1, and 2 h (G: 0.5, 1, and 2 h). The area under the curve (AUC) of OGTT was calculated as Equation 2:


$$\text{AUC of OGTT }=\;0.\text{25}\times \left({\text{G}}_{0\text{h}}+{\text{G}}_{0.\text{5h}}\right)+\;0.\text{ 25}\times \left({\text{G}}_{0.\text{5h}}+{\text{G}}_{\text{1h}}\right)+\;0.\text{ 5}\times \left({\text{G}}_{\text{1h}}+{\text{G}}_{\text{2h}}\right).$$ (2)

After an overnight 12 h fast (water *ad libitum*), mice were anesthetized by intraperitoneal injection of Zoletil (55 mg/kg) and xylazine hydrochloride (5 mg/kg). Blood was collected via orbital sampling, allowed to clot at room temperature for 2 h, and centrifuged at 2,000 rpm for 10 min to obtain serum for biochemical assays. Mice were euthanized by cervical dislocation, and liver, caecum and pancreas were rapidly excised. Portions of tissues were fixed in 4% paraformaldehyde for histopathology, whereas the remaining samples were snap-frozen in liquid nitrogen and stored at −80 °C until further analysis. The study was conducted in accordance with the Guide for the Care and Use of Laboratory Animals issued by the National Institutes of Health (Publication No. 85-23, revised 1985). All experimental procedures were reviewed and approved by the Institutional Animal Care and Use Committee of Jiangsu Vocational College of Medicine (approval number: SYLL-2024-710).

### 2.6 Homeostasis model assessment (HOMA) indices

Fasting insulin (FINs) in serum was quantified according to the manufacturer's instructions (Wuhan Chundu Biotechnology Co., Ltd., China) using an Enzyme-Linked Immunosorbent Assay (ELISA) kit. Homeostasis model assessment (HOMA) integrates FBG and FINs to estimate the balance between glucose metabolism and insulin homeostasis (27), including β-cell function (HOMA-β), insulin resistance (HOMA-IR) and insulin sensitivity (HOMA-IS). Indices were calculated as follows (28) Equations 3–5:


HOMA− ​​β​​ = 20 × FINs (mU/L)/(FBG (mmol/L)−3.5) (3)

$$\begin{array}{lll} \text{HOMA}-\text{IR} & = & \text{FINs}\left(\text{mU}/\text{L}\right)\;\times \text{FBG}\left(\text{mmol}/\text{L}\right)/\text{22}.\text{5} \end{array}$$ (4)

$$\begin{array}{lll} \text{HOMA}-\text{IS} & = & \text{1}/\left(\text{FINs}\left(\text{mU}/\text{L}\right)\;\times \text{FBG}\left(\text{mmol}/\text{L}\right)\right) \end{array}$$ (5)

### 2.7 Serum and hepatic biochemical measurements

Serum was prepared as described in Section 2.2. Liver tissues were rinsed with physiological saline on ice to remove residual blood, and 0.10 g of tissue was minced and homogenized in 0.90 mL saline (1:9, w/v). Homogenates were centrifuged at 4,000 rpm for 10 min, and supernatants were collected for downstream assays. TC, TG, LDL-c, HDL-c and glycated serum protein (GSP) in serum and hepatic supernatants were determined using commercial kits (Nanjing Jiancheng Bioengineering Institute), following the manufacturers' protocols.

### 2.8 Gut microbiota profiling

Caecal contents (0.10 g per mouse) were collected, and total microbial DNA was extracted using the QIAamp DNA Stool Mini Kit (Qiagen, Hilden, Germany). Amplification of the V3-V4 region was executed with primers 341 F (5′-CCTAYGGGRBGCASCAG-3′) and 806 R (5′-GGACTACNNGGGTATCTAAT-3′). Polymerase Chain Reaction (PCR) amplicons were separated on 2% agarose gels, purified using the AxyPrep DNA Gel Extraction Kit, and quantified fluorometrically. Libraries were sequenced on an Illumina NovaSeq platform (PE250) (Illumina, San Diego, CA, USA). Raw reads were subjected to quality control and normalization before downstream analyses. After quality filtering and chimera removal, a total of 2,676,468 high-quality clean reads were obtained from 36 samples, with an average of 74,346 reads per sample (range: 42,573–102,845; Supplementary Table S2). Community composition was profiled based on Operational Taxonomic Unit (OTU) clustering and taxonomic annotation. Spearman correlation analysis (R software, ver. 4.1.0) (R Foundation for Statistical Computing, Vienna, Austria) was used to assess associations between glycaemia-related parameters and genera, visualized as heatmaps; correlation networks were constructed in Cytoscape (ver. 3.9.0) (Cytoscape Consortium, San Diego, CA, USA) with edges retained for strong associations (|*r*| > 0.58).

### 2.9 Quantification of caecal short-chain fatty acids (SCFAs)

For each SCFAs (acetic, propionic, isobutyric, butyric, isovaleric and valeric acid), 100 μL of the standard solution was transferred into a 10 mL volumetric flask and brought to volume with anhydrous diethyl ether to generate single-standard stock solutions. Stocks were serially diluted to generate six calibration levels, which were analyzed by gas chromatography (GC) to construct standard curves (peak area vs. concentration). The detailed calibration ranges, regression equations, and correlation coefficient (R^2^) for each SCFA are summarized in Supplementary Table S3. Caecal contents (100 mg) were homogenized in deionized water at 1:10 (w/v), acidified with phosphoric acid, and vortexed for 2 min. SCFAs were extracted with anhydrous diethyl ether, filtered through a 0.22 μm nylon syringe filter, and analyzed by GC (Shimadzu, Kyoto, Japan).

GC conditions: HP-INNOWAX capillary column (30 m × 0.25 mm × 0.25 μm); high-purity nitrogen as carrier gas at 1.0 mL/min; injection volume 1 μL; injector 260 °C; detector 260 °C; split ratio 10:1. Oven program: 100 °C for 1 min, ramp at 5 °C/min to 200 °C, hold for 2 min.

### 2.10 Statistical analysis

Data are presented as mean ± SD. Normality of data distribution was assessed using the Shapiro–Wilk test. Differences among multiple groups were evaluated using the nonparametric Kruskal–Wallis test. The complete Kruskal–Wallis test statistics (H values, degrees of freedom, and *p*-values) for evaluated indices in Figures are detailed in Supplementary Table S4. When the Kruskal–Wallis test indicated a statistically significant overall difference, Dunn's *post hoc* test was subsequently performed for pairwise comparisons. Bonferroni correction was applied to all multiple pairwise comparisons. All *p* values reported in the manuscript represent Bonferroni-adjusted *p* values. Therefore, the significance threshold of *p* < 0.05 indicated in the figure legends refers to the Bonferroni-adjusted level of statistical significance. Exact adjusted *p* values are reported in the Section 3 wherever applicable. For figures involving multiple-group comparisons, the notation “different letters indicate statistically significant differences among groups” was used, with all such pairwise significance determinations based on Bonferroni-adjusted *p* values. For the longitudinal endpoints (body weight and FBG), cross-sectional comparisons were performed across five treatment groups at four distinct time points. Therefore, the Bonferroni correction for these analyses was applied over a total of 20 independent tests (5 × 4), resulting in an adjusted significance threshold of α = 0.05/20 = 0.0025. As a sensitivity analysis, we also repeated the multiple comparisons using the Benjamini–Hochberg False Discovery Rate (FDR) correction; the statistical outcomes were consistent with those obtained from the Bonferroni correction. All statistical analyses were performed using SPSS 16.0 (IBM, New York, USA). The sample size (*n* = 6 per group) was determined based on established conventions in the field and our prior experience with this T2DM mouse model. To further justify this, we performed a retrospective *post-hoc* power analysis based on FBG at week 4 (mean difference between PSQ-H and Model: 8.62 mmol/L; pooled SD: 2.25 mmol/L). This yielded a large effect size (Cohen's d = 8.62 / 2.25 = 3.83). Using a two-sided α = 0.05, the analysis confirmed that *n* = 6 per group provides statistical power exceeding 95%.

## 3 Results

### 3.1 The morphology and *in vitro* release of PSQ

**Figure 2:**
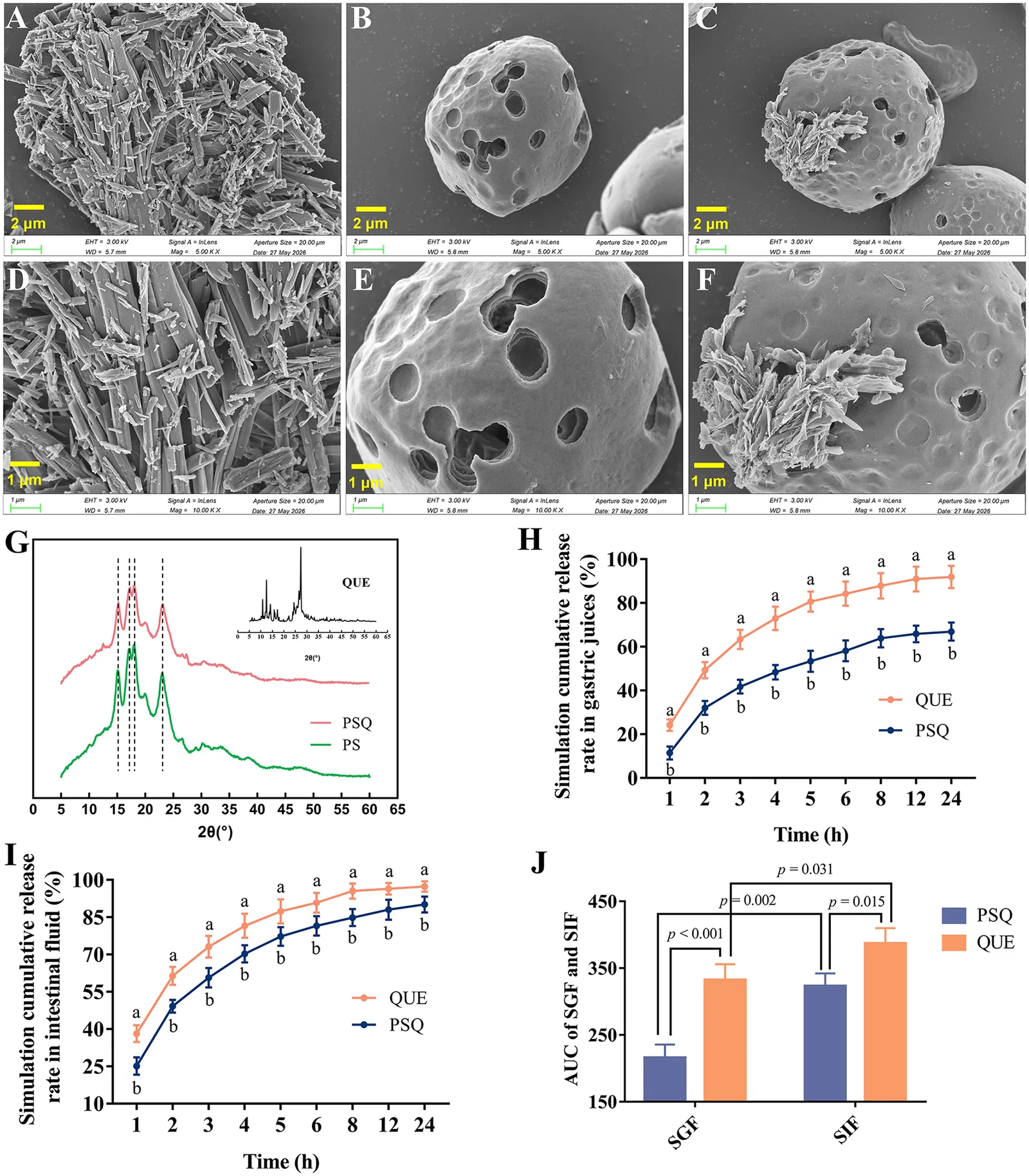
Characterization of PSQ and their *in vitro* release behavior. SEM images of QUE **(A, D)**, PS **(B, E)**, and PSQ **(C, F)** at 5,000 × **(A–C)** and 10,000 × **(D–F)**. Scale bars, 2 μm **(A–C)** and 1 μm **(D–F)**. XRD patterns of QUE, PS, and PSQ **(G)**. Cumulative release profiles of PSQ in SGF **(H)** and SIF **(I)**. AUC of SGF and SIF over 6 h, AUC was calculated from cumulative release (%) vs. time (h), with units expressed as % × h. A higher AUC value indicates greater overall release during the 6 h period. **(J)**. Within the same release medium, different letters indicate a statistically significant difference between PSQ and free QUE (*p* < 0.05).

Under the observation of SEM, QUE (Figures 2A, D) presented a rod-like form. PS (Figures 2B, E) has a hollow structure with numerous pores inside. This structure can adsorb QUE, providing protection or sustained-release effects. As shown in Figures 2C, F, the loading amount of QUE was significantly increased. It was distributed not only around the pore openings and inside the pore diameter, but also more widely distributed on the PS surface. Notably, although the SEM images obtained at magnifications of × 5,000 and × 10,000 showed QUE-associated material around the pore openings and on the particle surfaces, higher-resolution imaging would be required to accurately distinguish and quantify the relative proportions of intraporously loaded and surface-adsorbed QUE. The EE and DLC of PSQ were determined by measuring free QUE remaining in the supernatant after adsorption (HPLC). The EE of PSQ was 68.3 ± 2.5%, and the DLC was 25.4 ± 0.9%.

XRD analysis was performed to investigate the effect of loading onto the crystalline state of QUE. The XRD patterns of QUE, PS, and PSQ are presented in Figure 2G. PS exhibited a typical A-type crystalline structure, with sharp diffraction peaks at approximately 2θ = 15.06°, 17.98°, 18.06°, and 23.02° (29). Crystalline QUE showed characteristic diffraction peaks at 10.78°, 12.44°, 14.14°, 15.80°, 17.20°, 24.38°, and 27.34°. In contrast, none of the characteristic peaks of crystalline QUE were detected in the PSQ complex, indicating that QUE had transitioned from a crystalline state to an amorphous or highly molecularly dispersed state following loading into the PS matrix. Moreover, compared with pristine PS, PSQ exhibited markedly reduced diffraction intensities and broader, less-defined peaks, suggesting that the incorporation of QUE partially disrupted the ordered arrangement of the starch chains and consequently decreased the relative crystallinity of PS. This solid-state transformation may be attributed to non-covalent interactions, particularly hydrogen bonding, between QUE and starch molecules. During adsorption, these interactions disrupted the crystal lattice of QUE and facilitated its molecular dispersion within the pores and on the surface of PS (30). Collectively, the XRD results provide structural evidence that the porous starch carrier effectively altered the original crystalline structure of QUE and stabilized it in a higher-energy amorphous state. Because amorphization is generally considered beneficial for maintaining supersaturation of poorly water-soluble compounds in gastrointestinal fluids, these findings provide additional structural support for the potential of PS-based loading to enhance the intestinal availability of QUE (22). However, we acknowledge that the XRD analysis was performed on freshly prepared PSQ samples, and we did not verify whether the amorphous state of QUE is maintained after exposure to simulated gastrointestinal fluids, where recrystallization could occur during the release process. Future studies incorporating XRD or differential scanning calorimetry (DSC) analysis of PSQ samples recovered after *in vitro* release experiments would be valuable to confirm the stability of the amorphous form under gastrointestinal conditions. The BET specific surface area of PS was 2.6096 m^2^/g, whereas that of PSQ decreased to 1.2293 m^2^/g, corresponding to a reduction of 52.9%. This pronounced decrease was attributed to the loading of QUE (Supplementary Table S1). Specifically, QUE incorporation increased the total mass of the composite and occupied or blocked part of the accessible pore structure of PS, thereby reducing the surface area available for nitrogen adsorption. Consistently, the pore volume decreased from 7.198 × 10^−3^ cm^3^/g for PS to 2.796 × 10^−3^ cm^3^/g for PSQ, while the average pore size decreased from 94.7405 to 32.132 nm. The substantial reduction in pore size suggests that QUE entered the porous network and partially obstructed the pore channels during adsorption. In addition, PSQ exhibited a larger grain diameter than PS. Taken together, the reductions in BET surface area, pore volume, and average pore size, together with the increase in grain diameter and the disappearance of the characteristic crystalline peaks of QUE, collectively confirm that QUE was successfully loaded onto the surface and into the pores of PS. In SGF and SIF containing 0.5% Tween 80 (Figures 2H, I), the cumulative release of free QUE was significantly higher than that of PSQ at all time points. In SGF, the cumulative release of free QUE at 2, 6, and 24 h was approximately 49.3%, 84.2%, and 91.9%, respectively, whereas that of PSQ at the corresponding time points was only approximately 32.0%, 58.1%, and 66.9%. In SIF, the cumulative release of free QUE at 6 and 24 h was approximately 90.8 and 97.3%, respectively, whereas that of PSQ was approximately 81.5 and 90.1%. To further quantify the release behavior of PSQ under different conditions, the areas under the 0–6 h release curves in (AUC of SGF and SIF) were calculated (Figure 2J). The AUC of PSQ in SIF was significantly higher than that in SGF (*p* = 0.002). In addition, the AUC of PSQ were significantly lower than those of free QUE in both SGF and SIF (*p* < 0.001 and *p* = 0.015, respectively). It should be noted that the absolute release rates presented here were obtained in the presence of 0.5% Tween 80 to maintain sink conditions, a standard practice for dissolution testing of poorly soluble compounds. The absolute values may differ under physiological conditions without surfactant; however, the relative differences between PSQ and free QUE, as well as the consistently higher release in SIF than in SGF, support the conclusion that PS encapsulation provides partial gastric protection and enhanced intestinal delivery.

### 3.2 Effects of PSQ on body-weight loss and glycaemic control in T2DM mice

**Figure 3:**
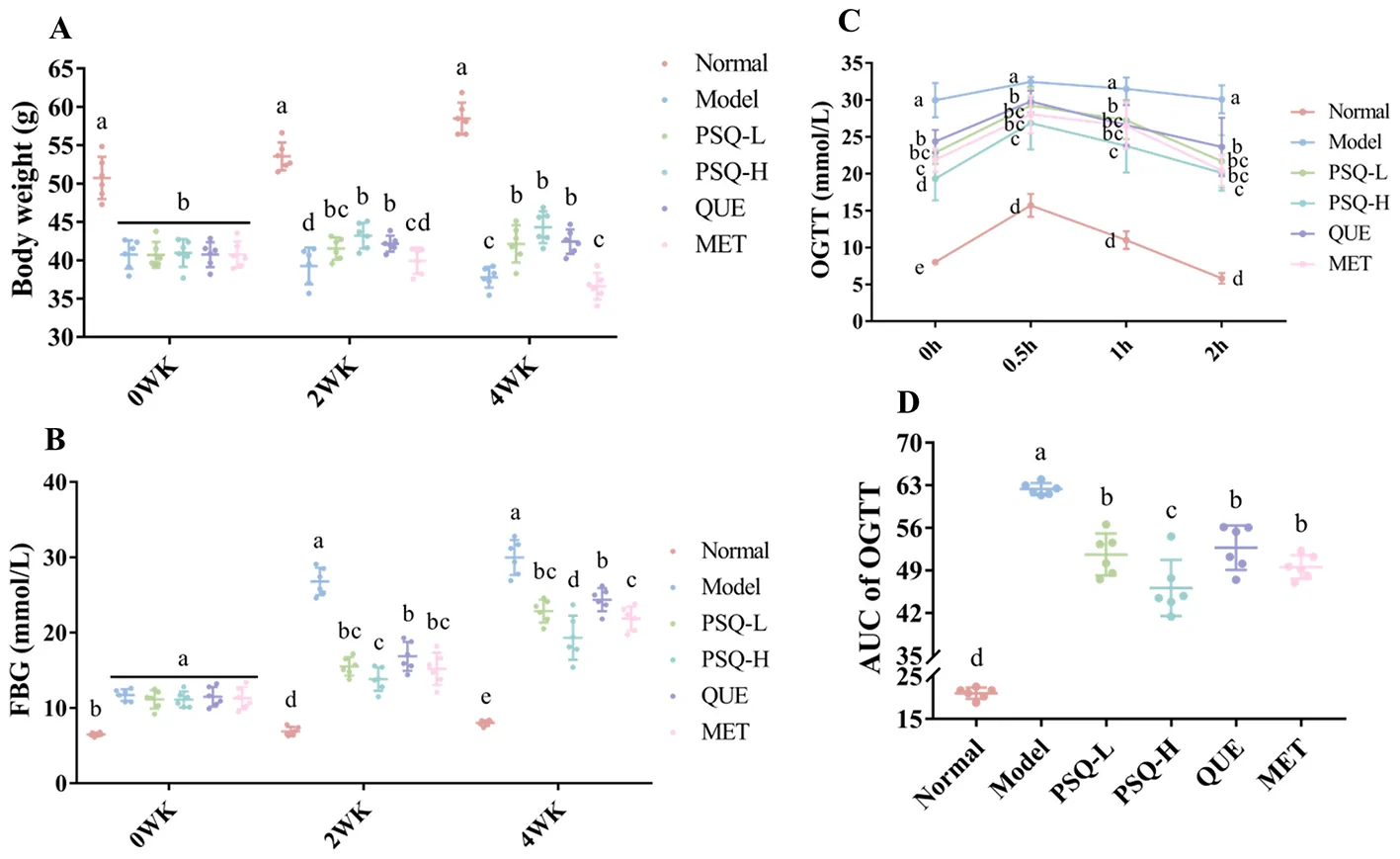
Effects of PSQ intervention during the experimental period. **(A)** Body weight, **(B)** FBG levels, **(C)** OGTT levels, and **(D)** AUC of OGTT. PSQ, porous starch microspheres encapsulated with quercetin; T2DM, type 2 diabetes mellitus; WK, week. Different superscript letters indicate statistically significant differences between the groups (*p* < 0.05).

Body-weight trajectories are shown in Figure 3A. At baseline (week 0), all model-derived groups exhibited lower body weight than the Normal cohort (*p* < 0.001), consistent with the weight-loss phenotype following diabetes induction. At week 2, body weight was significantly higher in the Normal (*p* < 0.001), PSQ-L (*p* = 0.03), PSQ-H (*p* < 0.001) and QUE (*p* = 0.007) groups compared with the Model group, with PSQ-H also exceeding the MET group (*p* = 0.003). At week 4, Kruskal-Wallis test revealed significant group differences in body weight (H = 29.361, df = 5, *p* < 0.001). Body weight in all non-Normal groups remained below that of Normal (*p* < 0.001); however, PSQ-L, PSQ-H and QUE groups showed significantly higher body weight than both the Model and MET groups (*p* < 0.001), indicating better preservation of body weight under PSQ treatment.

Longitudinal changes in FBG are summarized in Figure 3B. At week 0, FBG was elevated in all diabetic groups compared with Normal (*p* < 0.001). At week 2, FBG in PSQ-L, PSQ-H, QUE and MET remained higher than Normal (*p* < 0.001), but was significantly lower than in the Model group (*p* < 0.05). At week 4, Kruskal-Wallis analysis confirmed a highly significant overall difference in FBG (H = 29.413, df = 5, *p* < 0.001). FBG in PSQ-L and PSQ-H remained above Normal (*p* < 0.001), whereas PSQ-L, PSQ-H, QUE and MET all showed lower FBG than Model (*p* < 0.001); notably, PSQ-H showed significantly lower FBG than PSQ-L (*p* = 0.003), QUE (*p* < 0.001) and MET (*p* = 0.026) at this time point, consistent with a stronger glycaemic benefit at the higher dose.

Glucose tolerance was assessed by OGTT (Figure 3C). Following oral glucose loading (2 g/kg body weight), blood glucose peaked at 0.5 h and declined thereafter across groups. At 0 h, glucose levels were lowest in Normal and highest in Model (*p* < 0.001). At 0.5 h, PSQ-L (*p* = 0.019), PSQ-H (*p* < 0.001), QUE (*p* = 0.048) and MET (*p* = 0.002) exhibited lower glucose than Model, and PSQ-H was lower than QUE (*p* = 0.031). At 1 and 2 h, glucose continued to decrease, yet remained higher in PSQ-L, PSQ-H, QUE and MET than in Normal (*p* < 0.001) while staying below Model (*p* < 0.05); PSQ-H also remained lower than QUE at 2 h (*p* = 0.029). Consistently, AUC of OGTT was reduced in PSQ-L, PSQ-H, QUE and MET relative to Model (*p* < 0.001), with PSQ-H showing a lower AUC than PSQ-L (*p* = 0.002), and QUE (*p* < 0.001; Figure 3D).

### 3.3 PSQ improves islet-function indices and serum biochemical parameters in T2DM mice

**Figure 4:**
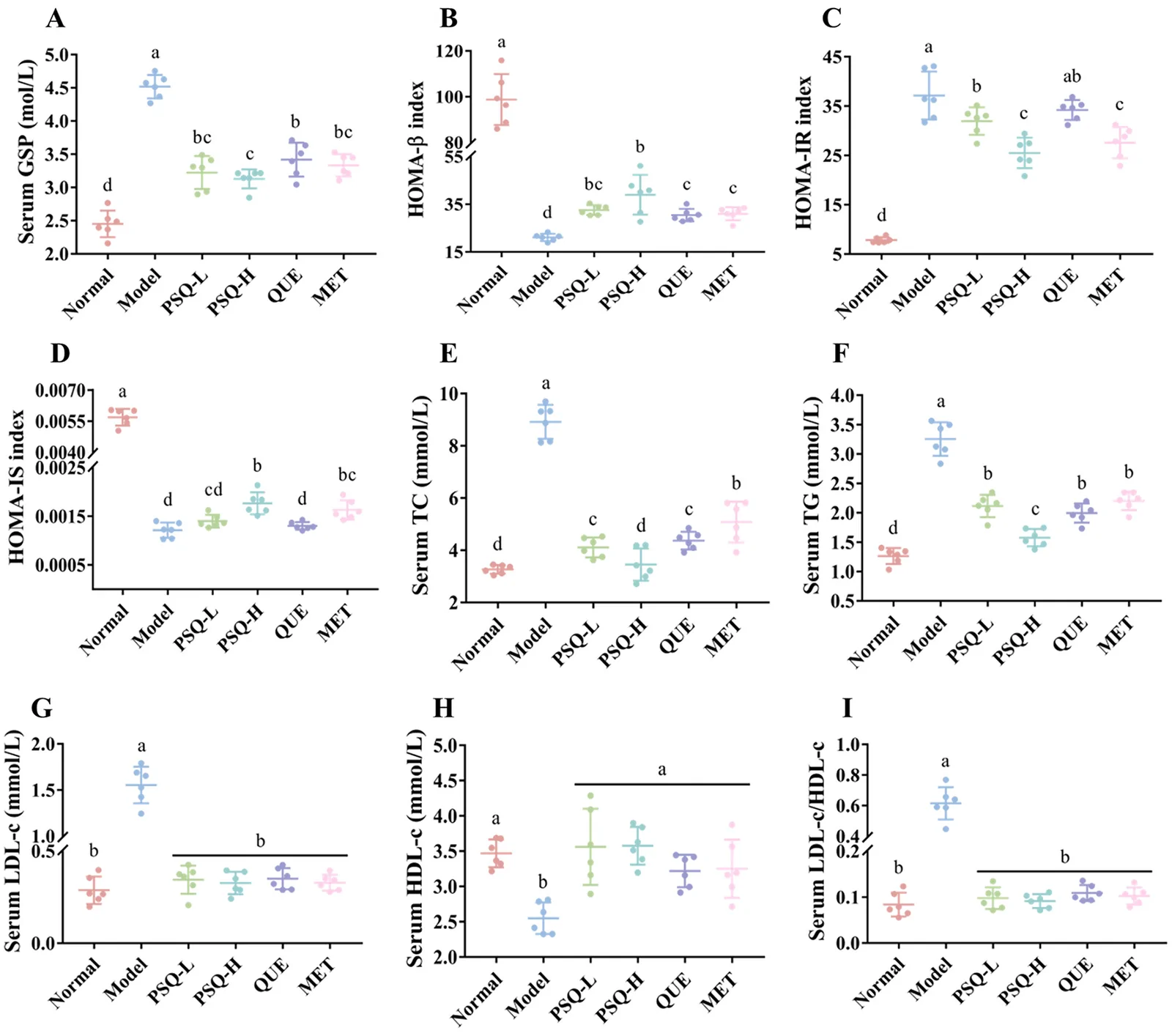
Pancreatic islet function correlation indices and serum biochemical indicators. **(A)** serum GSP, **(B)** HOMA-β, **(C)** HOMA-IR, **(D)** HOMA-IS, **(E)** serum TC, **(F)** serum TG, **(G)** serum LDL-c, **(H)** serum HDL-c, and **(I)** serum LDL-c/HDL-c.

GSP is shown in Figure 4A. Compared with the Model group, GSP levels were significantly reduced in all other groups (*p* < 0.001), and PSQ-H further decreased GSP level relative to QUE (*p* = 0.019), indicating a larger improvement in an intermediate-term marker of glycaemic exposure at the higher PSQ dose.

HOMA-β increased across all non-Model groups relative to Model (*p* < 0.05), with PSQ-H exceeding QUE and MET (Figure 4B). In parallel, HOMA-IR was lower in Normal (*p* < 0.001), PSQ-L (*p* = 0.006), PSQ-H (*p* < 0.001) and MET (*p* < 0.001) than in Model, and PSQ-H was further reduced compared with PSQ-L (*p* = 0.001) and QUE (*p* < 0.001; Figure 4C). For HOMA-IS, Normal remained significantly higher than all other groups (*p* < 0.001), whereas PSQ-H (*p* < 0.001) and MET (*p* = 0.003) increased HOMA-IS relative to Model (Figure 4D).

Because T2DM is frequently accompanied by dyslipidaemia, serum lipid-related indices were quantified (Figures 4E–I). Serum TC was decreased in all non-Model groups compared with Model (*p* < 0.001; Figure 4E); TC was lower in PSQ-L than in MET (*p* = 0.004), and PSQ-H further reduced TC relative to PSQ-L (*p* = 0.041), QUE (*p* = 0.006) and MET (*p* < 0.001). A similar pattern was observed for serum TG (Figure 4F), with PSQ-H exhibiting lower TG than PSQ-L (*p* < 0.001), QUE (*p* = 0.001) and MET (*p* < 0.001). Consistently, LDL-c was elevated in the Model group (*p* < 0.001; Figure 4G), whereas HDL-c was reduced (*p* < 0.05; Figure 4H). The LDL-c/HDL-c ratio, used as an integrated proxy of lipid-related risk, was significantly lower in all non-Model groups than in Model (*p* < 0.001; Figure 4I).

### 3.4 PSQ attenuates hepatic dyslipidaemia in T2DM mice

**Figure 5:**
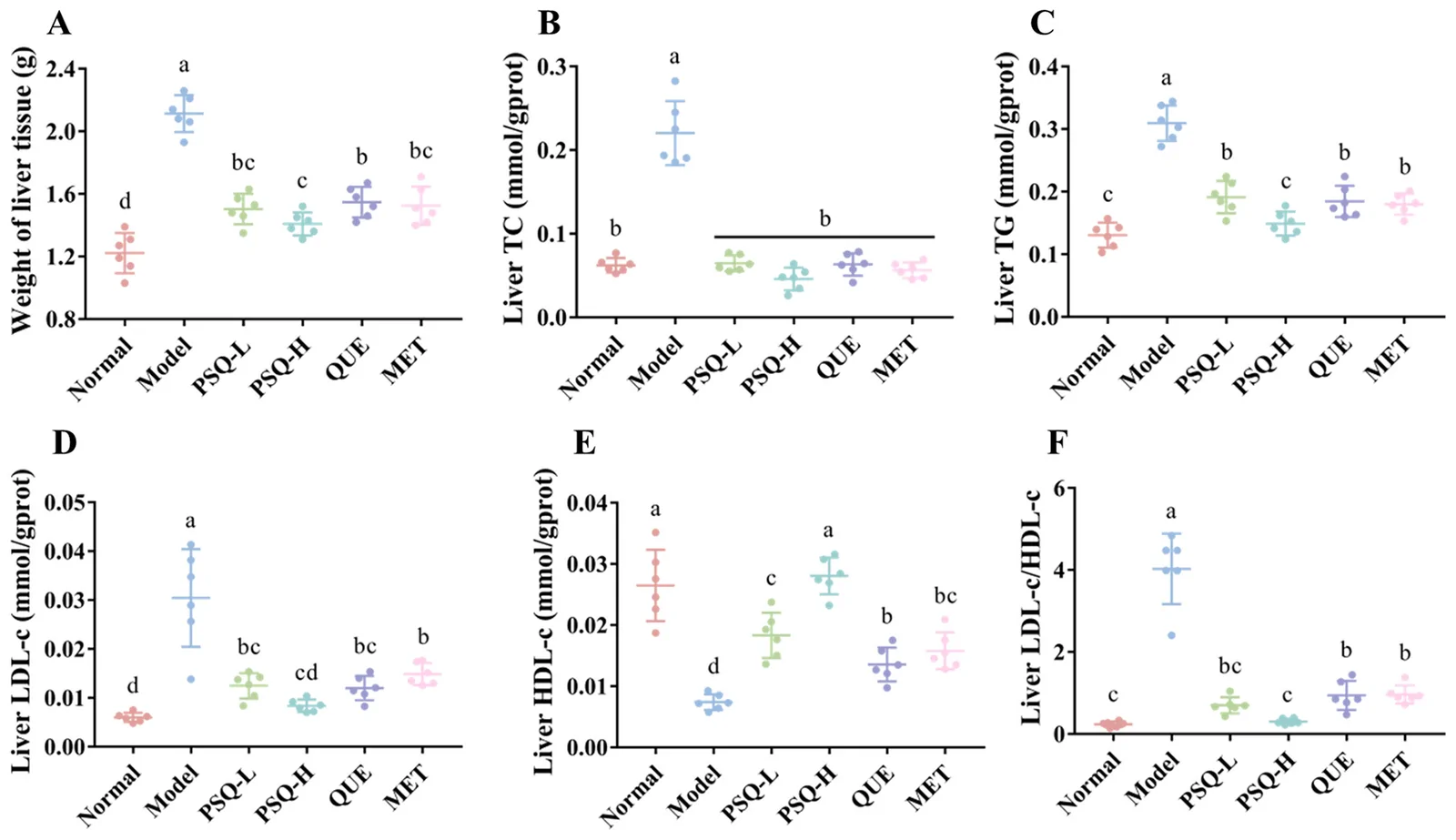
Biochemical indicators of liver tissue. **(A)** weight of liver tissue, **(B)** liver TC, **(C)** liver TG, **(D)** liver LDL-c, **(E)** liver HDL-c, and **(F)** liver LDL-c/HDL-c.

Dysregulated lipid metabolism in T2DM is frequently accompanied by enhanced hepatic lipid synthesis and accumulation, which can be coupled to cellular stress responses and tissue injury. Accordingly, we quantified liver weight and hepatic lipid-related indices across groups (Figures 5A–F). Liver weight is shown in Figure 5A. Compared with the Model group, liver weight was significantly lower in all other groups (*p* < 0.001), and PSQ-H further reduced liver weight relative to QUE (*p* = 0.034). Hepatic lipid parameters are summarized in Figures 5B–D. Relative to Model, hepatic TC, TG and LDL-c were decreased in all non-Model groups (*p* < 0.001). Notably, PSQ-H lowered hepatic TG compared with PSQ-L (*p* = 0.003), QUE (*p* = 0.012) and metformin (MET; *p* = 0.026; Figure 5C), and hepatic LDL-c was also lower in PSQ-H than in MET (*p* = 0.018; Figure 5D). Conversely, hepatic HDL-c was reduced in the Model group relative to all other groups (*p* < 0.05), and PSQ-H increased hepatic HDL-c compared with PSQ-L, QUE and MET (*p* < 0.001; Figure 5E). Consistently, the hepatic LDL-c/HDL-c ratio, used here as an integrated proxy of lipid imbalance, was reduced in all non-Model groups compared with Model (*p* < 0.001), with PSQ-H further lower than QUE (*p* = 0.01) and MET (*p* = 0.008; Figure 5F).

### 3.5 PSQ remodels gut microbial composition and increases caecal SCFAs

**Figure 6:**
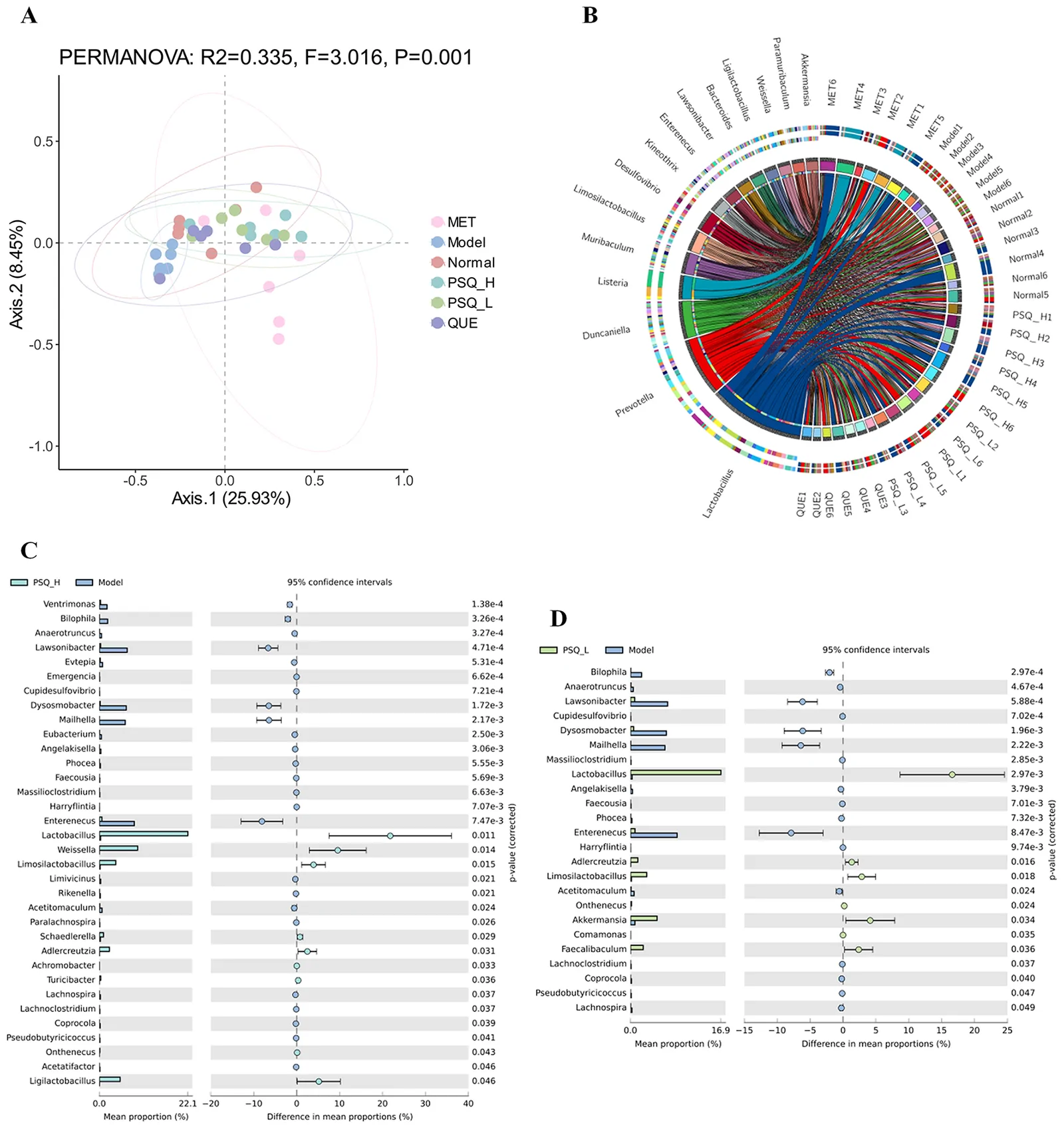
Effects of PSQ treatment on intestinal microbial communities in the cecum: **(A)** Principal coordinate analysis (PCoA) with Bray–Curtis distances. Axis1 and Axis2 explained 25.93 and 8.45% of the total variance, respectively, accounting for 34.38% of the cumulative variance, **(B)** the dominant microbiota in each sample at the genus level, **(C, D)** Extended error bar plot showing significant differences in intestinal microbiota at the genus level: **(C)** PSQ-H *vs*. Model; **(D)** PSQ-L *vs*. Model.

To capture global microbiota differences across groups, principal coordinate analysis (PCoA) with Bray–Curtis distances was applied to community profiles. Normal and Model samples were well-separated, and both PSQ-L and PSQ-H communities also diverged from the Model cluster (Figure 6A), consistent with a PSQ-associated shift in microbiota structure. A Circos overview summarized the dominant genera across samples (Figure 6B), with the top 15 taxa including *Lactobacillus, Prevotella, Duncaniella* and *Listeria*. Genus-level differential abundance analysis (STAMP) identified taxa underlying group differences. Relative to Model, PSQ-H enriched *Lactobacillus* (*p* = 0.011), *Weissella* (*p* = 0.014), *Adlercreutzia* (*p* = 0.031), *Limosilactobacillus* (*p* = 0.015), and *Ligilactobacillus* (*p* = 0.046), while depleting *Enterenecus, Mailhella, Dysosmobacter, Lawsonibacter, Bilophila* and *Ventrimonas* (*p* < 0.001; Figure 6C). PSQ-L similarly increased *Lactobacillus* (*p* < 0.001), *Adlercreutzia* (*p* = 0.016), *Limosilactobacillus* (*p* = 0.018), *Akkermansia* (*p* = 0.034) and *Faecalibaculum* (*p* = 0.036), and reduced *Enterenecus, Mailhella, Dysosmobacter, Lawsonibacter, Bilophila* and *Anaerotruncus* (*p* < 0.001; Figure 6D). Notably, both doses produced concordant directional shifts, enrichment of *Lactobacillus, Adlercreutzia* and *Limosilactobacillus* together with depletion of *Enterenecus, Mailhella, Dysosmobacter, Lawsonibacter* and *Bilophila*, suggesting a consistent microbiota response to PSQ.

**Figure 7:**
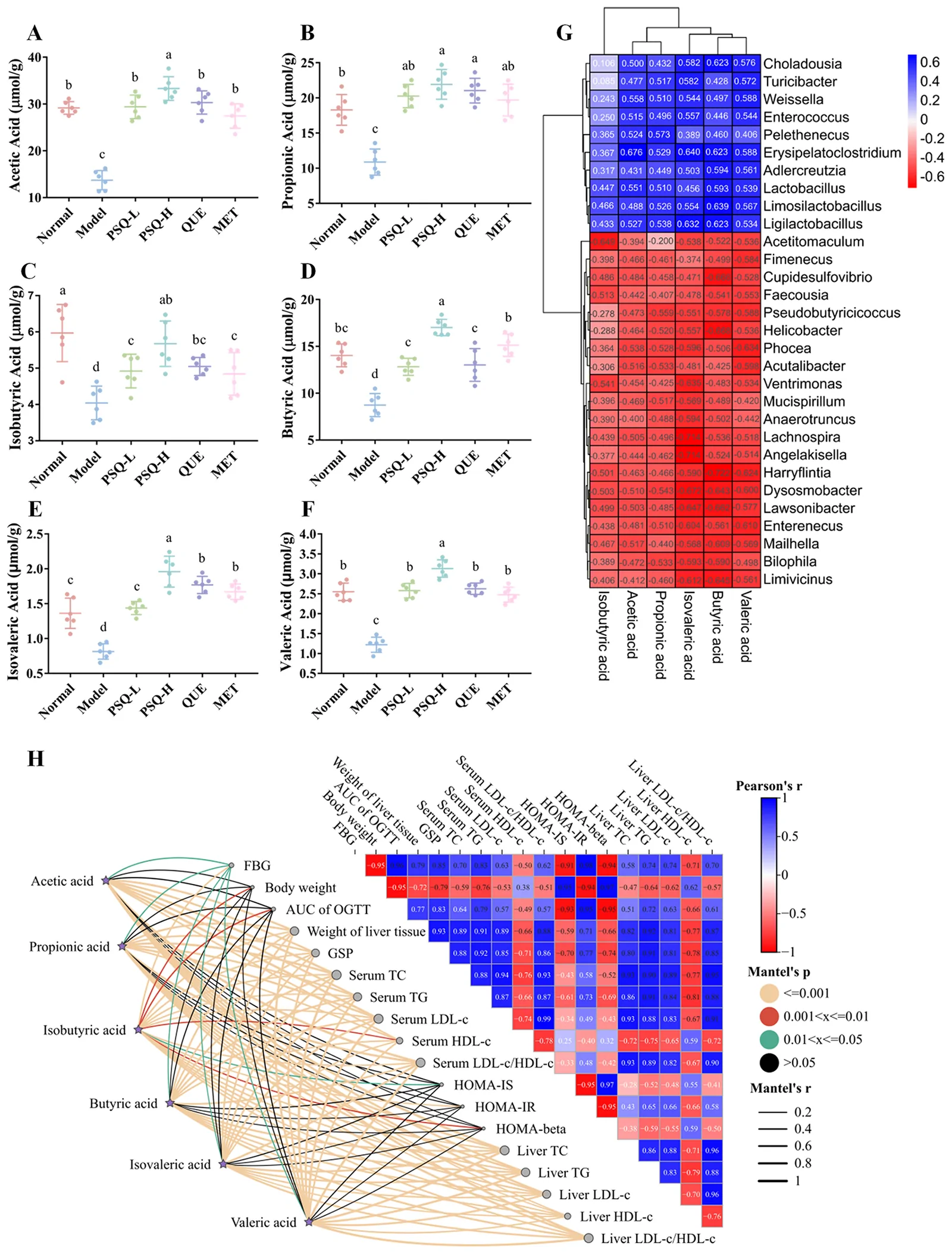
Effects of PSQ intervention on the contents of SCFAs in the cecum of mice: **(A)** Acetic acid, **(B)** Propionic acid, **(C)** Isobutyric acid, **(D)** Butyric acid, **(E)** Isovaleric acid, **(F)** Valeric acid, **(G)** Hierarchical clustering heatmap showing Spearman correlations between bacterial genera and short-chain fatty acids (SCFAs). The color scale represents the Spearman correlation coefficient *r*, with blue indicating positive correlations and red indicating negative correlations. Color intensity is proportional to the strength of the correlation, **(H)** Mantel test analysis of the associations between hypoglycemic parameters and SCFAs. The color scale represents the Mantel correlation coefficient *r*, with blue indicating positive correlations and red indicating negative correlations. Color intensity is proportional to the strength of the correlation.

Because SCFAs are major fermentation products of dietary carbohydrates, caecal acetic, propionic, isobutyric, butyric, isovaleric and valeric acids were quantified (Figures 7A–F). Compared with Model, both PSQ-L and PSQ-H increased all measured SCFAs (*p* < 0.001). With the exception of propionic acid, PSQ-H further elevated acetic (*p* = 0.006), isobutyric (*p* = 0.025), butyric (*p* < 0.001), isovaleric (*p* < 0.001) and valeric acids (*p* < 0.001) relative to PSQ-L, indicating a dose-dependent enhancement of SCFAs output. To link taxa to metabolites, genus-SCFAs correlations were examined (Figure 7G). *Lactobacillus* and *Limosilactobacillus* correlated positively with all SCFAs, whereas *Enterenecus, Mailhella, Dysosmobacter, Lawsonibacter* and *Bilophila* showed negative correlations. Mantel testing further indicated significant associations between multiple hypoglycaemia-related parameters, including FBG, liver weight, serum GSP and a panel of serum/hepatic lipid indices, and each SCFAs (Figure 7H).

**Figure 8:**
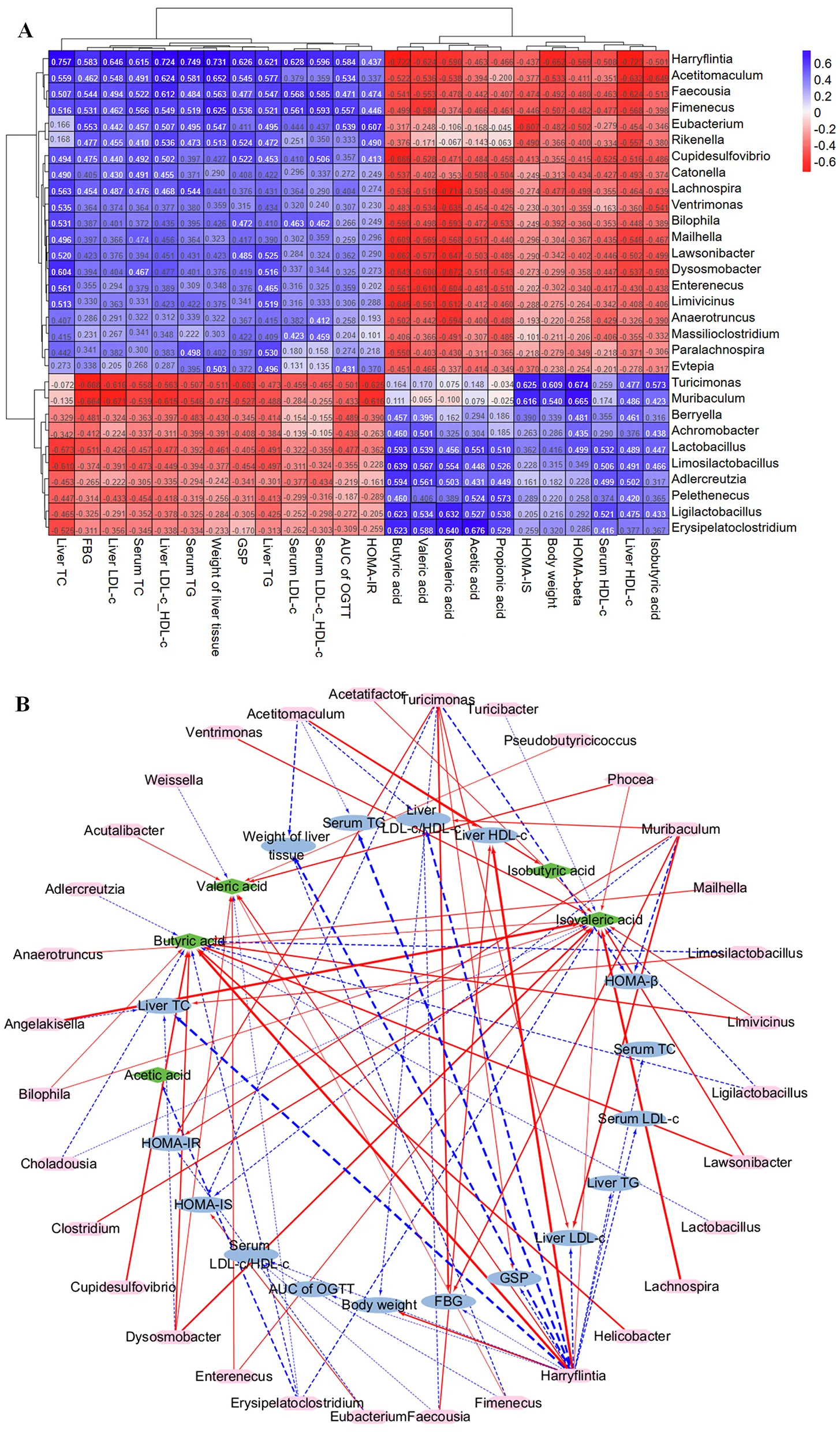
Hierarchical clustering analysis **(A)** and visualization of the correlation network **(B)** using Spearman correlation of hypoglycemic parameters and representative bacterial genus. In **(A)**, Hierarchical clustering heatmap showing Spearman correlations between bacterial genera and glycemic parameters. The color scale represents the Spearman correlation coefficient *r*, with blue indicating negative correlations and red indicating negative correlations. Color intensity is proportional to the strength of the correlation. In **(B)**, Correlation network among hypoglycemic parameters, representative bacterial genera, and SCFAs. Powder-blue, light-pink, and apple-green nodes represent hypoglycemic parameters, representative bacterial genera, and SCFAs, respectively. Blue dotted lines indicate significant negative correlations (*r* > 0.58, adjusted *p* < 0.01), whereas red solid lines indicate significant positive correlations (*r* < −0.58, adjusted *p* < 0.01). Line thickness is proportional to the absolute value of the correlation coefficient.

We next assessed correlations between representative differential genera and metabolic readouts (Figure 8A). Overall, *Lactobacillus* tracked positively with multiple SCFAs, HOMA-IS, body weight, HOMA-β and HDL-c related measures, while showing negative associations with HOMA-IR, AUC of OGTT, FBG, serum GSP and several dyslipidaemia-related indices; *Limosilactobacillus* displayed a similar pattern. Conversely, *Enterenecus, Mailhella, Dysosmobacter* and *Bilophila* were positively associated with hyperglycaemia- and dyslipidaemia-related indices and negatively associated with SCFAs and HDL-c measures. To emphasize strong associations, a cytoscape network was constructed using edges with |*r*| ≥0.58 (Figure 8B). *Harryflintia* exhibited robust positive correlations with hepatic TC, serum TG, liver weight, hepatic LDL-c/HDL-c, hepatic LDL-c, serum LDL-c, serum GSP, hepatic TG and serum TC (*r* = 0.757, 0.749, 0.731, 0.724, 0.646, 0.628, 0.626, 0.621 and 0.615, respectively), and strong negative correlations with valerate, body weight, butyrate and hepatic HDL-c (*r* = −0.624, −0.652, −0.722 and −0.723, respectively). *Turicimonas* correlated positively with HOMA-β, HOMA-IS and body weight (*r* = 0.674, 0.625 and 0.609) and negatively with serum GSP, hepatic LDL-c, HOMA-IR and FBG (*r* = −0.603, −0.616, −0.625 and −0.668). *Muribaculum* correlated positively with HOMA-β and HOMA-IS (*r* = 0.665 and 0.616) and negatively with hepatic LDL-c/HDL-c, HOMA-IR, FBG and hepatic LDL-c (*r* = −0.615, −0.616, −0.644 and −0.672).

## 4 Discussion

T2DM is a prevalent endocrine metabolic disorder that imposes a substantial and growing burden on human health. Chronic exposure to a high-fat/high-sugar diet can precipitate elevated fasting glycaemia, impaired oral glucose tolerance and perturbed islet function; in parallel, the persistent hyperglycaemic milieu characteristic of T2DM is frequently accompanied by hepatic dyslipidaemia. QUE has been proposed to mitigate these derangements, potentially by reshaping gut microbial composition and altering microbe-derived metabolite profiles, which may associate with alleviating hepatic metabolic dysfunction and improving hyperglycaemia-associated phenotypes. Nevertheless, the translational potential of QUE remains constrained by pharmaceutic limitations, most notably its limited stability and poor aqueous solubility, which collectively result in low oral bioavailability and restrict broader biomedical application. To address this delivery bottleneck, we formulated PSQ using corn-derived PS as the carrier. In addition to the porous-starch-based system used in this study, various other delivery strategies have been reported in the literature to overcome quercetin's poor oral bioavailability. Alizadeh and Ebrahimzadeh comprehensively reviewed both structural modification and drug delivery strategies-including cyclodextrin inclusion complexes, phospholipid complexes, solid dispersions, nanoemulsions, nanoparticles, and liposomes-aimed at improving quercetin's water solubility and bioavailability (31). Tomou et al. (32) further provided an overview of nanoformulation strategies, highlighting that lipid-based nanocarriers (liposomes, nanostructured lipid carriers, solid lipid nanoparticles) and polymer-based nanocarriers (micelles, hydrogels) have been widely explored to improve quercetin's oral absorption and sustained release. Zhao et al. (33) similarly summarized that delivery systems such as microparticles, solid dispersions, phospholipid complexes, and hydrogels have demonstrated stronger pharmacological effects in preclinical studies compared with free quercetin. Each of these systems has its own advantages and trade-offs in terms of manufacturing complexity, cost, scalability, and regulatory feasibility. Compared with these platforms, our PS-based approach offers a complementary advantage-using a widely available, low-cost, food-grade starch carrier that is biodegradable, scalable, and suitable for functional-food applications, which may facilitate its eventual translation into practical use.

The QUE remaining in PSQ after incubation in simulated gastric fluid may comprise two fractions: QUE that remained unreleased because of physical entrapment within the pores and QUE that failed to enter the dissolved phase because of its inherently poor aqueous solubility. The former may be released in the intestine as the starch matrix undergoes degradation, whereas the latter may dissolve as the increased intestinal pH improves QUE solubility. These findings suggest that the residual QUE may still be available for further release and utilization during subsequent intestinal transit. The cumulative release of QUE from PSQ in simulated gastric fluid reached 58.1% at 6 h, indicating that a proportion of QUE was released in the stomach because the formulation lacked a sealing layer. Therefore, the improved *in vivo* effects of PSQ may be partly attributable to an increased amount of QUE reaching the intestine. Nevertheless, gastric protection and increased intestinal delivery are not mutually exclusive. PSQ reduced gastric QUE release from approximately 84.2% for free QUE to 58.1% after 6 h, thereby allowing a greater proportion of QUE to remain available for subsequent intestinal delivery. Analysis of the area under the *in vitro* release curve showed that the release AUC of PSQ in SIF was significantly higher than that in SGF, indicating that a greater amount of QUE was released from PSQ under intestinal conditions. This result is consistent with the findings of Zhao et al., (34) who reported that quercetin incorporated into broken-rice porous starch exhibited significantly lower release than free quercetin in both simulated gastric and intestinal fluids. In addition, XRD analysis showed that QUE was present in PSQ in an amorphous state, which may provide greater apparent solubility than its crystalline form. The cumulative release of QUE from PSQ in SIF reached 90.1% at 24 h, further indicating that the porous starch carrier enabled the release of most of the loaded QUE under intestinal conditions. Collectively, the enhanced effects of PSQ may arise through multiple mechanisms: partial gastric protection reduces premature gastric release of QUE, porous starch loading increases the amount of QUE available for intestinal delivery, and conversion to the amorphous state improves its availability under intestinal conditions.

By the end of PSQ intervention, body weight was significantly restored in both PSQ-L and PSQ-H groups relative to the Model cohort, consistent with an attenuation of the diabetes-associated weight-loss phenotype. Notably, MET did not induce a comparable weight rebound at either 2 or 4 weeks, and the MET group exhibited a lower mean body weight than the Model group at week 4, a pattern that may reflect MET-mediated inhibition of intestinal glucose absorption and utilization (35). With respect to glycaemic control, FBG, a widely used clinical indicator, declined markedly after 2 and 4 weeks of PSQ intervention, following an overall trajectory comparable to the MET positive control. Importantly, PSQ-H achieved lower FBG levels than QUE at both time points, indicating that PS encapsulation potentiates the antihyperglycaemic effect of QUE *in vivo*, consistent with the *in vitro* release profile showing restrained gastric liberation and enhanced intestinal release (Figures 2H, I). However, because FBG reflects a single fasting-time snapshot and is subject to physiological fluctuation, reliance on FBG alone may be insufficient for robust evaluation; accordingly, complementary measures of glucose handling are typically required. As reported by Jiao et al., (36) OGTT-derived metrics, including the AUC of OGTT, together with GSP can provide additional resolution for assessing T2DM-associated perturbations in glucose homeostasis. In our study, OGTT curves for QUE, PSQ-L and PSQ-H lay between those of Normal and Model animals, whereas the AUC of OGTT was significantly lower in PSQ-H than in PSQ-L and QUE. Collectively, these findings indicate that, relative to free QUE, high-dose PSQ more effectively ameliorates hyperglycaemia-related phenotypes in T2DM mice.

GSP provides a relatively stable readout of mean glycaemic exposure over the preceding 2–3 weeks and is less susceptible to short-term glucose fluctuations, thereby serving as an informative integrative index of glucose metabolism and β-cell function (37). In this study, GSP was significantly lower in the PSQ-H group than in the QUE group, a pattern that aligned with the changes observed in fasting blood glucose and AUC of OGTT, and collectively suggests that high-dose PSQ more effectively attenuates hyperglycaemia-associated phenotypes in T2DM mice. β-cell dysfunction is widely regarded as a central driver of T2DM pathogenesis, and sustained impairment of β-cell capacity can, in turn, exacerbate insulin resistance, manifested as diminished insulin responsiveness in peripheral tissues such as liver and skeletal muscle. Accordingly, quantitative evaluation of β-cell function and insulin sensitivity constitutes a critical component of therapeutic assessment in T2DM (38). HOMA is commonly used for this purpose, with HOMA-β, HOMA-IR and HOMA-IS reflecting β-cell function, insulin resistance and insulin sensitivity, respectively. Here, PSQ-L did not differ significantly from QUE across HOMA-β, HOMA-IR and HOMA-IS, whereas PSQ-H showed significant differences from QUE for all three indices, indicating a more pronounced improvement in islet-related metrics at the higher PSQ dose. Beyond hyperglycaemia and islet impairment, T2DM is frequently accompanied by dyslipidaemia, including elevated triglycerides and cholesterol, which can aggravate hepatic and systemic injury (39). In line with this, both serum and hepatic lipid abnormalities were evident in the T2DM model; notably, PSQ-H improved serum TC and TG more strongly than PSQ-L and QUE, suggesting superior modulation of lipid dysregulation. The liver is a central hub of lipid homeostasis, and high-fat/high-sugar feeding can induce hepatic injury, which may reduce both the abundance and affinity of insulin receptors on hepatocyte membranes, thereby perturbing insulin signaling and promoting insulin resistance (40). Consistent with this result, QUE, PSQ-L and PSQ-H each partially improved hepatic indices, with PSQ-H outperforming QUE for hepatic TG, hepatic HDL-c and the hepatic LDL-c/HDL-c ratio. Collectively, these findings indicate that, relative to free QUE, high-dose PSQ confers more substantial improvements in GSP, HOMA-derived indices and serum/hepatic biochemical abnormalities in T2DM mice.

The human gut harbors a microbial community on the order of trillions of bacteria, which coexists with the host through structured symbiotic relationships that collectively shape metabolic homeostasis. Increasing evidence indicates that maintaining compositional stability and functional balance of the gut microbiota is critical for normal glucose regulation. Although the first two PCoA axes accounted for 34.38% of the total variance, with the remaining variation likely attributable to inherent individual heterogeneity, PERMANOVA confirmed that treatment effects significantly explained 33.5% of the overall community variation (*R*^2^ = 0.335, *p* = 0.001). This validates the robustness and statistical reliability of our group separation. At the genus level, *Lactobacillus, Adlercreutzia* and *Limosilactobacillus* are frequently discussed as taxa linked to metabolic health. Lin et al. (41) reported that betulinic acid increased the relative abundance of short-chain fatty acid associated taxa, including *Lactobacillus*, together with microbial metabolites, concomitant with improvements in hyperglycaemia-related phenotypes. Chen et al. (42) further showed that galacto-oligosaccharide supplementation enriched *Lactobacillus* and *Adlercreutzia* relative to a high-fat diet group, and that *Adlercreutzia* was positively associated with bile salt hydrolase activity, an axis implicated in lipid metabolism. Jeon et al. (43) demonstrated that an extract of *Psidium guajava* L. leaves fermented with *Limosilactobacillus* significantly improved fasting glycaemia and alleviated insulin resistance in diabetic mice. In the present study, both PSQ-L and PSQ-H were accompanied by increased relative abundances of *Lactobacillus, Adlercreutzia* and *Limosilactobacillus*; taken together with the glycaemic improvements observed, these data suggest that enrichment of these genera were correlated with the attenuation of hyperglycaemia-associated phenotypes in T2DM mice.

SCFAs represent one of the most prominent classes of gut microbial metabolites and are generated predominantly through anaerobic fermentation of carbohydrates that escape digestion and absorption in the upper gastrointestinal tract (44). Beyond serving as an important energy source for both gut microbes and intestinal epithelial cells, SCFAs are involved in intestinal homeostasis by modulating luminal pH, supporting epithelial growth, and constraining the expansion of opportunistic pathogens. A growing body of epidemiological and mechanistic evidence further links SCFAs to insulin sensitivity and glucose homeostasis, implying that increased SCFAs availability may help reduce the risk of metabolic disorders such as obesity, T2DM and cardiovascular disease. Notably, individual SCFAs exhibit site- and function-specific profiles: acetic acid, the most abundant SCFA, acts predominantly in adipose tissue (45), propionic acid is closely tied to colonic and hepatic metabolism (46), whereas butyric acid has been implicated in pancreatic functional regulation (47). In the present study, after 4 weeks of intervention with QUE, PSQ-L or PSQ-H, caecal concentrations of acetic, propionic, isobutyric, butyric, isovaleric and valeric acids were all significantly increased in T2DM mice; moreover, acetic, butyric, isovaleric and valeric acids were higher in PSQ-H than in QUE, indicating that PS encapsulation may potentiate the rise in caecal SCFAs during QUE-based intervention. Given that SCFAs production is tightly shaped by microbiota composition, prior work supports a microbiota-SCFA-phenotype linkage: Park et al. (48) reported that *Lawsonibacter* was positively associated with fasting blood glucose yet negatively associated with SCFAs levels, whereas Mo et al. (49) showed that fermented dietary fiber derived from soy sauce residue reduced the abundance of *Bilophila* while increasing SCFAs in T2DM mice, concomitant with improved diabetic phenotypes. Consistent with these observations, our correlation analysis revealed significant negative associations between *Lawsonibacter*/*Bilophila* and multiple SCFAs (acetic, propionic, isobutyric, butyric, isovaleric and valeric acids). Collectively, these data suggest that microbiota remodeling accompanied by elevated SCFAs output was associated with the improved hyperglycaemia-related phenotypes observed under PSQ-H treatment. To further interrogate whether PSQ-associated glycaemic improvements co-vary with microbiota shifts, we performed hierarchical clustering and genus-level correlation analyses linking microbial taxa to glycaemia-related readouts. *Lactobacillus*, a canonical probiotic genus, has been reported to confer multiple host benefits when administered at appropriate dose (50). Consistently, Yue et al. (51) described a significant negative association between *Lactobacillus* abundance and FBG, alongside a close relationship with SCFAs levels. In line with these observations, PSQ intervention in our study was accompanied by enrichment of *Lactobacillus* together with increased caecal SCFAs, co-occurring with improved hyperglycaemia-associated indices, suggesting that this genus is statistically associated with the metabolic benefits observed under PSQ treatment. Beyond enrichment of putatively beneficial taxa, PSQ administration was also associated with depletion of genera such as *Lawsonibacter* and *Bilophila*, and these shifts paralleled improvements in FBG, serum GSP, AUC of OGTT and HOMA-β, consistent with a microbiota phenotype coupling in the T2DM model. Moreover, correlation-network analysis identified *Turicimonas* and *Muribaculum* as taxa that were strongly positively correlated with HOMA-β and HOMA-IS, while showing strong negative correlations with hepatic LDL-c, HOMA-IR and FBG. These associations nominate *Turicimonas* and *Muribaculum* as candidate microbial signatures that track with key features of T2DM progression.

These observations are consistent with the findings reported by Hu et al. (52) and Chen et al. (53). Recent studies have also highlighted the capacity of food-derived polysaccharides, such as *Pueraria lobata* polysaccharides, which remodel gut microbiota and activate hepatic FGF21 signaling, to exert metabolic benefits via the microbiota-host axis (54). The present findings, together with our *in vitro* release data, illustrate that the benefit of PS encapsulation is integrative. The starch carrier provides a food-compatible platform that shifts quercetin liberation toward the intestinal phase. This shift, in turn, allows the flavonoid to more effectively engage downstream microbiota, SCFA responses that associate with improved glycaemic control. A key distinction from polysaccharide-focused studies is worth emphasizing here. In those studies, the polymer itself resists digestion and acts as a direct microbial substrate. We acknowledge that even digestible starches such as porous starch may exert independent biological effects beyond their role as a delivery vehicle. Recent evidence indicates that starch fine molecular structures can selectively modulate gut microbial communities and promote the production of specific short-chain fatty acids (55), and a human trial demonstrated that SCFAs concentrations increased most during digestible starch supplementation (56). Nevertheless, the structural features of starch-particularly its porosity and enzymatic susceptibility-deserve consideration when interpreting its role in the gastrointestinal environment. Shen et al. (57) demonstrated that lipid complexation reduces rice starch digestibility and boosts SCFAs production via selective modulation of the gut microbiota, with starch-lipid complexes significantly increasing acetate and propionate concentrations during *in vitro* colonic fermentation. A more recent study further examined the digestive characteristics of porous starches from different cereals, showing that pore-dominant starches (corn and buckwheat) underwent an “inside-out” enzymatic hydrolysis pathway, and buckwheat starch with porous structures achieved a 28.19% reduction in glycemic index through increased resistant starch content (58). Additionally, dietary starch with higher amylose content and lower digestibility has been shown to increase digesta mass, provide a favorable environment for microbial growth in the distal gut, and promote SCFAs production, along with enrichment of beneficial genera such as *Bifidobacterium* and *Lactobacillus* (59). These findings are relevant to our study because porous starch, despite being digestible, possesses a unique pore structure that influences its enzymatic hydrolysis kinetics. While PS itself is hydrolyzed in the upper gut and does not act as a resistant starch or prebiotic fiber, its porous architecture provides the physical basis for quercetin encapsulation and controlled release, which constitutes the central mechanism we propose. Moreover, porous starch itself has been shown to influence gut microbial composition in T2DM models (60), suggesting that the carrier may have intrinsic biological activity. Therefore, the present findings should be interpreted as the integrated outcome of the PSQ composite, encompassing both the porous starch carrier and the encapsulated quercetin, rather than the effects of quercetin alone. Future studies incorporating PS-only and physical mixture (QUE + PS without encapsulation) control groups are necessary to systematically deconvolute the contributions of gastric protection, carrier-related effects, and potential synergistic interactions between PS and QUE.

A limitation of this study is the absence of a PS only control group, which would help distinguish whether the observed effects arise primarily from PS mediated gastric protection of QUE or from independent actions of PS itself. An additional consideration is that porous starch, upon enzymatic digestion, generates glucose, which could theoretically introduce an extra caloric/glycemic load in a diabetic model. However, the dose dependent superiority of PSQ-H over PSQ-L indicates that any such glucose load is outweighed by the anti-hyperglycemic effect of QUE. Collectively, the *in vitro* release profiles, the dose dependent efficacy of PSQ, and the superiority of PSQ over QUE support that PS encapsulation enhances QUE delivery and bioactivity. Nevertheless, a PS only control group would be required to formally quantify the net impact of the carrier, and future studies incorporating such a group are warranted to further dissect these mechanisms. Notably, approximately 32% of QUE was released from PSQ during the first 2 h in simulated gastric fluid, a time frame that more closely reflects the typical gastric residence time in mice (approximately 1–3 h). This suggests that premature release during the physiologically relevant gastric-transit period is relatively limited. In contrast, the cumulative release reached 58.1% after 6 h, indicating that a larger proportion of QUE is released only after prolonged gastric exposure, which may overestimate gastric release under more realistic transit conditions. Nonetheless, the 6-h cumulative value remains useful as a secondary indicator of the overall release potential of the formulation in the absence of a sealing layer. While this proof-of-concept study demonstrated that PS can partially reduce gastric release of QUE compared with the free compound, the substantial release observed at 6 h may constrain the overall delivery efficiency and therapeutic efficacy of PSQ. Therefore, future studies will encapsulate PSQ with a sodium alginate/nanocellulose/carboxymethyl chitosan interpenetrating polymer network to create a gastro-resistant sealed system for enhanced delivery targeting the gut microbiota. We also acknowledge that our statistical analyses for longitudinal endpoints (body weight and FBG) were based on cross-sectional comparisons at individual time points with Bonferroni correction, rather than repeated-measures ANOVA or mixed-effects models that formally account for within-subject correlations across time. While this approach is commonly used in the field for small-sample non-normal data and our conclusions are supported by consistent patterns across multiple time points, we recognize that mixed-effects models would provide a more rigorous framework for evaluating time × treatment interactions. Future studies will incorporate such methods to better characterize longitudinal treatment effects. We acknowledge that the initial screening for diabetes model establishment relied on a single FBG measurement after STZ injection, without a second confirmation at 48 h later. Although the stability of the diabetic state was subsequently confirmed by FBG monitoring and OGTT results, we recognize that confirmation at multiple time points during the initial screening would be a more rigorous approach and should be adopted in future studies. Several limitations should be considered when interpreting the SCFAs data. First, caecal SCFAs concentrations reflect the net balance between production, absorption, and metabolism, and thus group differences indicate relative changes without distinguishing the contribution of each individual process (61). Second, isobutyric acid and isovaleric acid are branched-chain SCFAs derived from amino acid fermentation and have distinct biological implications from carbohydrate-derived straight-chain SCFAs (acetic acid, propionic acid, and butyric acid) (62). Although branched-chain SCFAs increased and correlated negatively with glycemic indices in this study, the increases in straight-chain SCFAs were more pronounced, suggesting that branched-chain SCFAs changes may be a secondary consequence of global microbiota remodeling. Third, this correlational study does not establish causation among microbiota, SCFAs, and glycemic control; future studies using fecal microbiota transplantation, antibiotic depletion, or SCFAs supplementation are needed to define causal directions (63). Additionally, the present study measured only SCFAs and did not assess other microbially derived metabolites, including bile acids, trimethylamine N-oxide (TMAO), and indole derivatives. This limited scope prevents us from determining whether these metabolites also participate in the metabolic improvements observed with PSQ treatment. Future investigations incorporating targeted metabolomic analyses of these additional metabolites will be required to provide a more comprehensive understanding of the microbiological mechanisms underlying PSQ-mediated host metabolic regulation (64). Moreover, the primary advance of this study lies in the demonstration that the delivery strategy based on PS can effectively protect QUE from degradation in simulated gastric fluid and promote its release in simulated intestinal fluid. These effects may be related to the improvement of anti-hyperglycemic efficacy in T2DM mice. This distinguishes our work from recent studies that focus on the intrinsic microbiota modulating properties of food derived polysaccharides (54). In the present study, the gut microbiota and SCFAs changes were presented as correlative evidence supporting improved intestinal delivery of QUE, rather than as an independent discovery of novel prebiotic agents. Therefore, we believe that the main contribution of this study lies in the verification of a practical and suitable packaging strategy for food use, which can overcome the oral bioavailability limitations of QUE, and the association between the microbiota and SCFAs further confirms this conclusion. The translational relevance of quercetin for T2DM management is further supported by human clinical evidence. In a randomized controlled trial, Mantadaki et al. (65) investigated quercetin supplementation (500 mg/day for 12 weeks) in patients with T2DM and found that quercetin was associated with a significant increase in mean telomere length, suggesting potential clinical benefits at the cellular level. Another randomized controlled trial demonstrated that 500 mg/day quercetin supplementation improved glycated hemoglobin, systolic blood pressure, lung function, sleep duration, anxiety levels, and overall quality of life in T2DM patients (66). In a large population-based study using NHANES data (2007–2018), dietary quercetin intake was inversely associated with the risk of diabetic nephropathy, with each unit increase in log-transformed quercetin intake corresponding to a 38.10% reduction in DN risk (OR: 0.619; 95% CI: 0.457–0.839) (67). A comprehensive review by Tomasz et al. (68) concluded that quercetin mitigates diabetes-related hormonal and metabolic disorders and reduces oxidative and inflammatory stress in both animal and human studies. Collectively, these clinical and epidemiological studies support the translational potential of quercetin as a dietary supplement for T2DM management. However, they also highlight the critical need for improved delivery strategies to overcome quercetin's poor oral bioavailability, a gap that our PSQ formulation aims to address. The consistency between our preclinical findings and the beneficial effects observed in human studies reinforces the relevance of our work for future translational research. Notably, based on Reagan-Shaw body surface area normalization, the 100 mg/kg dose of PSQ-H in mice translates to a human equivalent dose (HED) of ~8.1 mg/kg (~486 mg/day for a 60 kg adult). This value closely aligns with the 500 mg/day regimen evaluated in clinical trials, underscoring the translational potential of the PSQ formulation.

## 5 Conclusion

This study systematically evaluated the efficacy of PSQ in ameliorating hyperglycaemia-associated phenotypes in T2DM. PSQ intervention significantly improved multiple glycaemic endpoints, including body weight, FBG, AUC of OGTT, GSP, and HOMA-derived indices (HOMA-β and HOMA-IR/IS). Dose-response comparisons further indicated that PSQ-H produced larger improvements than PSQ-L across key glycaemic parameters (FBG, AUC of OGTT and HOMA-IR/IS) and selected lipid-related readouts in serum and liver (including serum TC, serum TG, hepatic TG and hepatic HDL-c), consistent with a dose-dependent benefit. In addition, PSQ administration was accompanied by enrichment of genera frequently linked to metabolic health (for example, *Lactobacillus, Adlercreutzia* and *Limosilactobacillus*) together with depletion of taxa such as *Lawsonibacter* and *Bilophila*, alongside a marked increase in caecal SCFAs. Correlation analyses additionally nominated *Turicimonas* and *Muribaculum* as taxa that track closely with multiple glycaemia-related indices, providing candidate microbial signatures for subsequent mechanistic interrogation.

## Data Availability

The original contributions presented in this study are included in the article/Supplementary material. Further inquiries can be directed to the corresponding author.
