# Supplementary material for: Porous-starch-encapsulated quercetin remodels the gut microbiota and elevates short-chain fatty acids, alleviating hyperglycaemia-associated phenotypes in T2DM mice
Source: Front Nutr. 2026 Sep 9;13:1851659. doi: 10.3389/fnut.2026.1851659 (PMC13599724; doi:10.3389/fnut.2026.1851659)
Supplement: Supplementary file 1 [file Data_Sheet_1.docx]

**Porous-starch-encapsulated quercetin remodels the gut microbiota and elevates short-chain fatty acids, alleviating hyperglycaemia-associated phenotypes in T2DM mice**

Peipei Kang^a^, Yongyan Zheng^b^, Shuman Song^a^, Yiyin Gao^b^, Yuanhong Song^b^, Huayang Wang^b^, Tao Jiang^a,*^, Xinran Xiang^b,*^

^a^ Luoyang Key Laboratory of Zebrafish Toxicology Research, Luoyang Key Laboratory of Transplantation and Immunological Studies for Haematological Diseases, Department of Clinical Laboratory, the First Affiliated Hospital, and College of Clinical Medicine of Henan University of Science and Technology, Luoyang, China, 471003.

^b^ Jiangsu Key Laboratory of Huaiyang Food Safety and Nutrition Function Evaluation, Jiangsu Collaborative Innovation Center of Regional Modern Agriculture & Environmental Protection, School of Life Science, Huaiyin Normal University, Huai’an 223300, China.

Table S1. BET surface area, pore volume, average pore size, and grain diameter of PS and PSQ.

| Sample | BET surface area (m^2^/g) | Pore volume (cm^3^/g) | Average pore size (nm) | Grain diameter (μm) |
| --- | --- | --- | --- | --- |
| PS | 2.6096 | 7.198×10^-3^ | 94.7405 | 11.019 |
| PSQ | 1.2293 | 2.796×10^-3^ | 32.132 | 11.112 |

Table S2 The number of reads per sample

| sample-id | non-chimeric |
| --- | --- |
| PSQ_L1 | 58287 |
| PSQ_L2 | 42573 |
| PSQ_L3 | 73855 |
| PSQ_L4 | 63049 |
| PSQ_L5 | 73604 |
| PSQ_L6 | 70343 |
| PSQ_H1 | 49726 |
| PSQ_H2 | 86244 |
| PSQ_H3 | 102845 |
| PSQ_H4 | 79454 |
| PSQ_H5 | 78753 |
| PSQ_H6 | 78512 |
| MET1 | 71439 |
| MET2 | 75174 |
| MET3 | 70580 |
| MET4 | 72882 |
| MET5 | 52093 |
| MET6 | 72561 |
| Model1 | 79027 |
| Model2 | 96614 |
| Model3 | 79110 |
| Model4 | 95130 |
| Model5 | 84780 |
| Model6 | 86473 |
| Normal1 | 83796 |
| Normal2 | 86827 |
| Normal3 | 71755 |
| Normal4 | 76184 |
| Normal5 | 67694 |
| Normal6 | 76148 |
| QUE1 | 69449 |
| QUE2 | 65473 |
| QUE3 | 85308 |
| QUE4 | 71544 |
| QUE5 | 64139 |
| QUE6 | 65043 |

Table S3 Calibration curve parameters for the quantification of SCFAs by gas chromatography

| SCFAs | Regression equations | Calibration range (μmol/L) | Correlation coefficient (R^2^) |
| --- | --- | --- | --- |
| Acetic Acid | y=2e+07x-7980.4 | 100-5000 | 0.9999 |
| Propionic Acid | y=5e+07x-22663 | 50-2500 | 0.9999 |
| Isobutyric Acid | y=7e+07x-21442 | 10-1000 | 1 |
| Butyric Acid | y=7e+07x-24223 | 50-2500 | 0.9999 |
| Isovaleric Acid | y=9e+07x+544.45 | 10-1000 | 0.9999 |
| Valeric Acid | y=9e+07x-4357.2 | 10-1000 | 1 |

Table S4 Complete Kruskal-Wallis test statistics for all measured parameters.

| Indicator | H values | Degrees of freedom (df) | *p*-value |
| --- | --- | --- | --- |
| Serum GSP | 26.905 | 5 | ＜0.001 |
| Serum TC | 27.859 | 5 | ＜0.001 |
| Serum TG | 31.096 | 5 | ＜0.001 |
| Serum LDL-c | 17.336 | 5 | 0.004 |
| Serum HDL-c | 17.82 | 5 | 0.003 |
| Serum LDL-c/HDL-c | 17.847 | 5 | 0.003 |
| Liver TC | 19.847 | 5 | 0.001 |
| Liver TG | 26.76 | 5 | ＜0.001 |
| Liver LDL-c | 28.249 | 5 | ＜0.001 |
| Liver HDL-c | 29.598 | 5 | ＜0.001 |
| Liver LDL-c/HDL-c | 30.772 | 5 | ＜0.001 |
| Body weight (Week 0) | 14.953 | 5 | 0.011 |
| Body weight (Week 2) | 24.006 | 5 | ＜0.001 |
| Body weight (Week 4) | 29.361 | 5 | ＜0.001 |
| FBG (Week 0) | 15.603 | 5 | 0.008 |
| FBG (Week 2) | 27.579 | 5 | ＜0.001 |
| FBG (Week 4) | 29.413 | 5 | ＜0.001 |
| OGTT (0 h) | 29.413 | 5 | ＜0.001 |
| OGTT (0.5 h) | 24.538 | 5 | ＜0.001 |
| OGTT (1 h) | 24.307 | 5 | ＜0.001 |
| OGTT (2 h) | 25.895 | 5 | ＜0.001 |
| AUC of OGTT | 28.127 | 5 | ＜0.001 |
| HOMA-IS | 28.877 | 5 | ＜0.001 |
| HOMA-IR | 28.877 | 5 | ＜0.001 |
| HOMA-β | 26.73 | 5 | ＜0.001 |
| Acetic acid | 23.435 | 5 | ＜0.001 |
| Propionic acid | 20.622 | 5 | 0.001 |
| Isobutyric acid | 19.162 | 5 | 0.002 |
| Butyric acid | 27.183 | 5 | ＜0.001 |
| Isovaleric acid | 29.342 | 5 | ＜0.001 |
| Valeric acid | 24.715 | 5 | ＜0.001 |
